# Towards Next-Generation Sequencing for HIV-1 Drug Resistance Testing in a Clinical Setting

**DOI:** 10.3390/v14102208

**Published:** 2022-10-07

**Authors:** Calesta Hui Yi Teo, Nurul Hannah Binte Norhisham, Ogestelli Fabia Lee, Siyu Png, Chean Nee Chai, Gabriel Yan, Julian Wei-Tze Tang, Chun Kiat Lee

**Affiliations:** 1 Yong Loo Lin School of Medicine, National University of Singapore, Singapore 117597, Singapore; 2 School of Life Sciences and Chemical Technology, Ngee Ann Polytechnic, Singapore 599490, Singapore; 3 School of Social Sciences, Nanyang Technological University, Singapore 639818, Singapore; 4 School of Life and Physical Sciences, PSB Academy, Singapore 039594, Singapore; 5 Department of Laboratory Medicine, National University Health System, Singapore 119228, Singapore; 6 Department of Medicine, National University Health System, Singapore 119228, Singapore; 7 Respiratory Sciences, University of Leicester, Leicester LE1 7RH, UK

**Keywords:** HIV-1, drug resistance, genotypic resistance testing, next-generation sequencing, high-throughput sequencing, Sanger, population sequencing

## Abstract

The HIV genotypic resistance test (GRT) is a standard of care for the clinical management of HIV/AIDS patients. In recent decades, population or Sanger sequencing has been the foundation for drug resistance monitoring in clinical settings. However, the advent of high-throughput or next-generation sequencing has caused a paradigm shift towards the detection and characterization of low-abundance covert mutations that would otherwise be missed by population sequencing. This is clinically significant, as these mutations can potentially compromise the efficacy of antiretroviral therapy, causing poor virologic suppression. Therefore, it is important to develop a more sensitive method so as to reliably detect clinically actionable drug-resistant mutations (DRMs). Here, we evaluated the diagnostic performance of a laboratory-developed, high-throughput, sequencing-based GRT using 103 archived clinical samples that were previously tested for drug resistance using population sequencing. As expected, high-throughput sequencing found all the DRMs that were detectable by population sequencing. Significantly, 78 additional DRMs were identified only by high-throughput sequencing, which is statistically significant based on McNemar’s test. Overall, our results complement previous studies, supporting the notion that the two methods are well correlated, and the high-throughput sequencing method appears to be an excellent alternative for drug resistance testing in a clinical setting.

## 1. Introduction

Human immunodeficiency virus type 1 (HIV-1, family *Retroviridae*, genus *Lentivirus*) has been a global health problem for many decades. In 2020, the Joint United Nations Program on HIV/AIDS reported over 37.7 million cases of human HIV-1 infections and 680,000 HIV/AIDS-related deaths worldwide [1]. Despite the absence of a complete cure for the disease, research has made great advances in providing treatment regimens to mitigate the transmission of the virus. The development of antiretroviral therapies (ART), such as the highly active antiretroviral therapy (HAART), has proved to be effective in the suppression of HIV replication [2], and numerous studies have shown that the use of HIV drug resistance tests to guide therapy is associated with improved treatment outcomes [3,4,5,6]. The global scale-up of HAART has also dramatically decreased the rates of AIDS-related mortality and morbidity [7].

Among all viruses, HIV has the highest and fastest replication rate, generating approximately 10 billion virions daily in treatment-naïve patients [8]. In addition, HIV has the highest mutation rate of approximately 1 mutation per genome per replication due to the lack of 3′-5′ proofreading exonuclease ability of the error-prone reverse transcriptase (RT) enzyme [9,10]. As a result, HIV is capable of rapid viral evolution. Most importantly, this may eventually lead to the emergence of HIV variants with drug-resistant mutations (DRMs).

DRMs are amino acid changes in well-characterized mutational hot spots associated with resistance to antiretroviral drugs [11,12,13]. Many DRMs have a reduced replicative fitness, which leads to the out-competition of DRMs by wild-type viruses because of the latter’s superior replication capacity [14]. The DRMs are therefore present as minority species at times. As the ART is only effective before the virus develops drug resistance, it is important to first screen for HIV drug resistance in patients initiating ART, followed by routine monitoring for resistance [15].

The transmission of HIV variants with DRMs can also have a significant impact on the effectiveness of the ART regimens and increase the risk of virological failure in treatment-naïve, HIV-infected patients [16,17,18]. Effective surveillance and screening for HIV DRMs in patients initiating ART and routine monitoring for resistance in ongoing ART regimens are, therefore, essential at the population level [15,19]. The selection of the ART, which is dependent on the genotypic result obtained from HIV drug resistance (HIVDR) testing, is thus of major significance.

Known as the “gold standard” for DNA sequencing [20,21], conventional genotypic resistance testing (GRT) using the Sanger sequencing technique, otherwise known as population sequencing (hereafter referred to as PS-GRT), has been recommended for HIVDR testing [22]. Since 2012, PS-GRT has been routinely employed by the National University Hospital (National University Health System, Singapore) to detect DRMs in HIV-infected patients. However, growing evidence has demonstrated that this conventional method is unable to reliably detect minority DRMs present at a level of less than 20% of the total virus population in the patient sample, i.e., a frequency level of less than 20% [12,23,24].

Several studies [25,26,27,28,29,30] have shown that minority DRMs are closely associated with treatment failures in patients initiating ART, which further highlights the impact of minority DRMs on clinical outcomes. In particular, this issue has been shown to be clinically significant in three settings: (1) the detection of non-nucleoside reverse-transcriptase-inhibitor (NNRTI)-resistant minority DRMs after exposure to single-dose nevirapine; (2) the detection of NNRTI-resistant minority DRMs prior to the initiation of a first-line NNRTI-based regime; and (3) the detection of CXCR4-using variants prior to treatment with CCR5 antagonists [31]. There is hence a need for a more sensitive method that can detect these minority DRMs to combat the risk of treatment failure.

Recently, a wealth of data demonstrating the use of the next-generation sequencing (NGS) based genotypic resistance tests, also known as high-throughput sequencing (hereafter referred to as HTS-GRT), for the detection of minority DRMs have been collected [23,24,32,33]. The significant cost reduction, higher throughput, and improved sensitivity [34] have led to the adoption of NGS as the preferred sequencing method, as opposed to the conventional sequencing. Since its inception, NGS has revolutionized the study of DRMs in HIV-infected patients in research settings, and laboratory-developed NGS-based tests are now increasingly being adopted by clinical laboratories for clinical-grade HIVDR testing [31]. In a study of 48 ART treatment-naïve patients, the mutation detection rate was found to be higher using NGS as compared to Sanger sequencing [24]. A separate study reported similar findings in patients who experienced ART interruption [33]. To date, the Sentosa SQ HIV genotyping assay (Vela Diagnostics, Singapore) is the only commercial NGS-based HIVDR platform that has been approved for in vitro diagnostic use by the United States Food and Drug Administration. It offers a semi-automated workflow and comes with a fully automated bioinformatics pipeline for drug resistance identification. By contrast, the laboratory-developed NGS-based tests have always been conducted manually and are laborious. Despite this, the latter remains invaluable to the clinical laboratories for its cost-effectiveness and flexibility, thereby enabling the affordable care of self-paying patients. On the other hand, there is a high cost associated with the commercially available platforms, which may potentially preclude patients from receiving the required standard of care.

Nevertheless, the utilization of the superior lower detection limit of the HTS-GRT (detection threshold < 20%) can be useful for selecting the most appropriate ART treatments for the patients by detecting the minority DRMs [35]. Here, we compare the clinical performance of a laboratory-developed HTS-GRT against a PS-GRT that has been routinely used for clinical HIVDR testing at our institution.

## 2. Materials and Methods

### 2.1. Clinical Samples and Ethics Approval

A total of 103 archived clinical samples, collected between 2015 and 2018, were included in the study. This study received local institutional ethics approval (from the National Healthcare Group Domain-Specific Review Board, reference number: DSRB/2018/00519).

### 2.2. Sample Processing

EDTA-anticoagulated blood was collected using the BD Vacutainer K2 EDTA blood collection tube. The blood collection tube was centrifuged at 3000× *g* for 10 min to separate the plasma from the blood cells. After concentration, 400 μL of plasma was subjected to viral RNA extraction using the QIAGEN EZ1 Virus Mini Kit v2.0 (QIAGEN, Hilden, Germany) with the BioRobot EZ1 Advanced XL workstation (QIAGEN) in line with the manufacturer’s protocol. The viral nucleic acid was eluted in 60 μL of elution buffer.

### 2.3. Population-Sequencing-Based Genotypic Resistance Test (PS-GRT)

PS-GRT was performed on all samples using a previously described method, with slight modifications [36]. Briefly, a single primer set (Table 1) was used to cover the entire protease (PR) and up to 395 codons in the reverse transcriptase (RT) of the *gag*-*pol* gene. A second primer set (Table 1) was included to cover up to 277 codons in the integrase (IN) of the *pol* gene. RT-PCR was carried out using the QIAGEN One- Step RT-PCR kit (QIAGEN). The cycling conditions used were initial reverse transcription (50 °C, 30 min) and denaturation (95 °C, 15 min) steps, followed by 50 cycles of amplification at 94 °C for 30 s, 51 °C for 30 s, and 72 °C for 1 min 30 s. Subsequent post-PCR and direct-sequencing workflows were conducted as previously described [36].

### 2.4. Manual Review of the Electropherograms

All electropherograms were manually reviewed using RECall [37]. During the review, the frequency level of the detected variants was recorded in a semi-quantitative manner at five frequency levels: 0%, <50%, 50%, >50%, and 100%. After review, the assembled contig sequences were uploaded to the Stanford HIV Drug Resistance Database using the HIVdb Program [38] for their drug resistance interpretation.

### 2.5. High-Throughput-Sequencing-Based Genotypic Resistance Test (HTS-GRT)

#### 2.5.1. High Fidelity Reverse Transcriptase Polymerase Chain Reaction

All samples tested on the PS-GRT were retested using HTS-GRT. Briefly, a single primer set (Table 1) was used to amplify the entire PR, entire RT, and up to 277 codons in the IN of the *gag*-*pol* gene. High-fidelity RT-PCR was carried out using the SuperScript™ III One-Step RT-PCR System with Platinum™ Taq High-Fidelity DNA Polymerase (Thermo Fisher Scientific, Waltham, MA, USA). The cycling conditions used were initial reverse transcription (50 °C, 30 min) and denaturation (94 °C, 2 min) steps, followed by 50 cycles of amplification at 94 °C for 15 s, 55 °C for 30 s, and 68 °C for 3 min. The PCR amplicons were separated by gel electrophoresis and visualized using the UV transilluminator to ensure that the expected targeted region (~3 kilobase pairs) was successfully amplified.

#### 2.5.2. Library Preparation and High-Throughput Sequencing

The DNA library preparation was carried out using the Nextera XT DNA Library Prep Kit (Illumina, San Diego, CA, USA) to generate indexed paired-end libraries from the PCR amplicons. The libraries were then loaded into the MiSeq sequencing system (Illumina) using the MiSeq Reagent Kit v2 Nano (2 × 150 reads).

### 2.6. Manual Review of the Binary Alignment Map Files

The binary alignment map (BAM) file contains the read mapping information in a compressed binary format. All BAM files were manually reviewed using Integrative Genomics Viewer (IGV, http://software.broadinstitute.org/software/igv/, accessed on 20 August 2022) to verify that the reads were correctly mapped based on five mapping quality metrics: a total read of ≥200, absence of strand bias, mapping quality of ≥30, quality value of ≥30, and 100% match in the compact idiosyncratic gapped alignment report (CIGAR). For this study, a 2% mutation detection threshold was used to identify a variant at any position. During the manual review, the frequency level of the detected variants was recorded. Like PS-GRT, the assembled contig sequences were uploaded to the Stanford HIV Drug Resistance Database using the HIVdb Program for their drug resistance interpretation [38].

### 2.7. Clinically Actionable Drug Resistance Mutations

We relied on the Stanford HIV Drug Resistance Database Version 9.1 [39] to distinguish the DRMs from the list of identified variants. Variants which conferred resistance (i.e., those that were assigned drug resistance mutation scores) to any of the four antiretroviral drug classes, including protease inhibitors (PI), nucleoside reverse transcriptase inhibitors (NRTIs), non-nucleoside reverse transcriptase inhibitors (NNRTIs), and integrase strand transfer inhibitors (INSTIs), were classified as clinically actionable DRMs.

### 2.8. Statistical Analysis

McNemar’s χ^2^ test, Deming regression, and Bland–Altman analyses were performed using the R version 3.6.0 to assess whether the differences in the numbers of detected variants and DRMs between PS-GRT and HS-GRT was statistically significant (with a *p*-value < 0.05 being statistically significant). Probit regression analysis was performed using R version 3.6.0 to estimate the 95% limit of detection (LoD) of the PS-GRT.

## 3. Results

### 3.1. HIV-1 Viral Load and Subtype Coverage

Among the 103 samples (Table 2), 44 samples had known viral loads, ranging from 2.77 to 6.78 log10 copies/mL, with a median of 4.80 log 10 copies/mL (interquartile range (IQR) of 4.35–5.20 log10 copies/mL). The main circulating subtypes within Singapore were found to be CRF01_AE (*n* = 70; 68%) and subtype B (*n* = 20; 19%), which is consistent with our earlier study [36]. The other strains detected (*n* = 13; 13%) were the subtypes C, G, and F2, as well as recombinant viruses, such as CRF33_01B, CRF07_BC, CRF48_01B, CRF51_01B, CRF52_01B, and CRF54_01B.

### 3.2. Agreement between PS-GRT and HTS-GRT

#### 3.2.1. McNemar’s χ^2^ Test

We performed McNemar’s χ^2^ test to assess whether the observed differences in the numbers of detected variants and DRMs between the PS-GRT and HTS-GRT was statistically significant. At the 2% mutation detection threshold (Table 3 and Table 4), a significant difference was observed for the PR variants (McNemar’s chi-squared = 45.021, df = 1, *p*-value < 0.05), RT variants (122.01, 1, <0.05), IN variants (23.04, 1, <0.05), and RT DRMs (69.014, 1, <0.05). No statistically significant difference was observed for the PR DRMs (1.3333, 1, 0.25) and IN DRMs (2.25, 1, 0.13). At the 20% mutation detection threshold (Table 3 and Table 4), no statistically significant difference was observed based on McNemar’s test.

#### 3.2.2. Spectrum of Detected Variants

**Figure 1:**
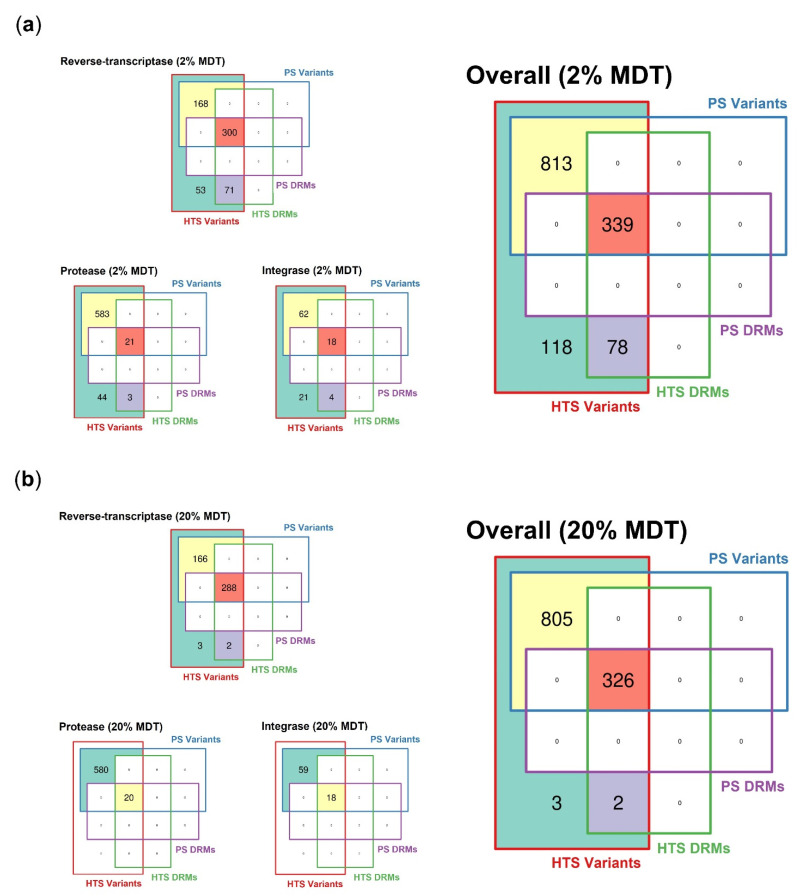
Agreement between PS-GRT and HTS-GRT in the analysis of 103 human immunodeficiency virus type 1 clinical samples: (**a**) Venn diagram of the total number of variants and DRMs detected by population sequencing and high-throughput sequencing at the 2% mutation detection threshold; (**b**) Venn diagram of the total number of variants and DRMs detected by population sequencing and high-throughput sequencing at the 20% mutation detection threshold. Note: HTS, high-throughput sequencing; PS, population sequencing; MDT, mutation detection threshold; DRM, drug resistance mutation.

In Figure 1a, we can see that the HTS-GRT detected a total of 1348 variants at the 2% detection mutation threshold. These variants were located in 86 unique codon positions within the HIV-1 *gag*-*pol* gene region (Table 3). By contrast, PS-GRT failed to detect a significant proportion of them (*n* = 196; 15%) in 68 unique codon positions at frequency levels ranging from 2% to 31%, with a median of 4% (IQR 2–9%). Among the missed variants, 40% (*n* = 78) were clinically actionable DRMs according to the Stanford HIV Drug Resistance Database.

**Figure 2:**
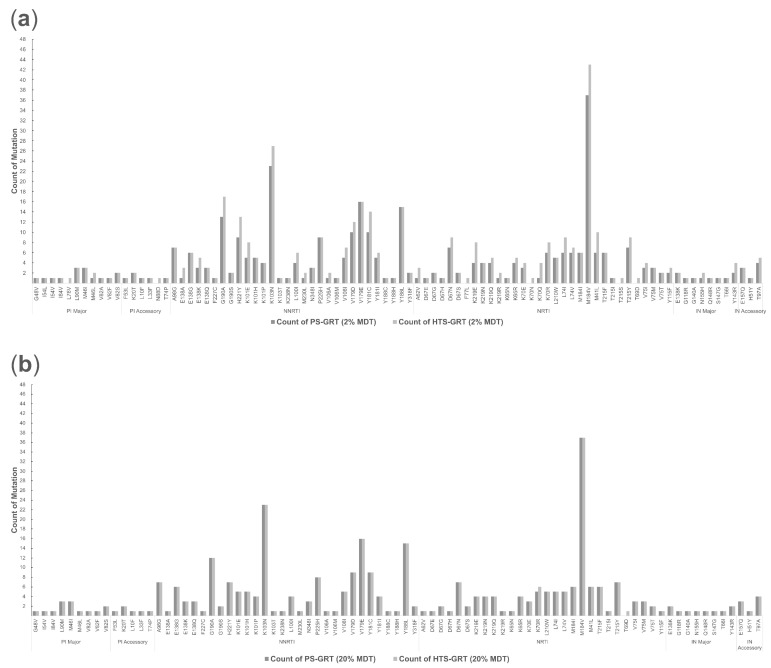
Frequency of HIV drug-resistant mutations that were detected using HTS-GRT and PS-GRT at the 2% and 20% mutation detection thresholds, respectively. (**a**) The 2% mutation detection threshold; (**b**) the 20% mutation detection threshold. Note: HTS-GRT, high-throughput-sequencing-based genotypic resistance test; PS-GRT, population-sequencing-based genotypic resistance test; PI, protease inhibitor; NRTI, nucleoside reverse transcriptase inhibitor; NNRTI, non-nucleoside reverse transcriptase inhibitor; IN, integrase; MDT, mutation detection threshold.

At the 20% mutation detection threshold (Figure 2a), 1131 variants were jointly detected by PS-GRT and HTS-GRT at 80 unique codon positions. Despite the fact that the generally accepted detection threshold for PS-GRT is 20%, it still failed to detect five RT variants, two of which were DRMs, at frequency levels ranging from 22% to 31%, with a median of 26% (IQR 23–31%). The frequency levels of the detected variants are summarized in Table 3.

#### 3.2.3. Spectrum of Detected Drug-Resistant Mutations

In Figure 1a, we can see that the HTS-GRT, at the 2% mutation detection threshold, detected a total of 417 DRMs. These DRMs were located in 57 unique codon positions within the *gag*-*pol* gene region of the HIV-1 genome (Table 4). Of the 417 DRMs, 24 were in the PR region, 371 were in the RT region, and 22 were in the IN region. For PR, the most frequent DRMs found in our study were M46I (*n* = 3; 13%) and L90M (*n* = 3; 13%), followed by K20T (*n* = 2; 8%), M46L (*n* = 2; 8%) and V82S (*n* = 2; 8%). For RT, the most frequent NRTI-associated DRM was M184V (*n* = 43; 12%), while the most frequent NNRTI-associated DRM was K103N (*n* = 27; 7%). For IN, the most frequent DRM was T97A (*n* = 5; 23%), followed by Y143R (*n* = 4; 18%).

Among the 417 DRMs that were identified by the HTS-GRT, 339 DRMs (81%) were also detected by PS-GRT, with their frequency levels ranging from 12% to 100% (median 100%; IQR 92–100%). As depicted in Figure 2a, HTS-GRT detected 78 additional DRMs, 3 of which were in the PR region, while 71 were in the RT region and 4 were in the IN region. The frequency levels of these DRMs ranged from 2% to 31%, with a median of 5% (IQR 3–11%). Among the 71 RT DRMs, the most frequent NRTI-associated DRM was M184V (*n* = 6; frequency level ranged from 2% to 19%), while the most frequent NNRTI DRMs were K103N (*n* = 4; frequency level ranged from 5% to 13%), Y181C (*n* = 4; frequency level ranged from 3% to 9%), G190A (*n* = 4; frequency level ranged from 2% to 13%), and H221Y (*n* = 4; frequency level ranged from 3% to 7%). Notably, K103N, Y181C, and M184V have been previously reported to be significantly associated with poor virologic suppression in the case of two EFV-based regimens, despite presenting at low frequency levels [12].

At the 20% mutation detection threshold (Figure 1b), a total of 326 DRMs were jointly detected by PS-GRT and HTS-GRT, 20 of which were in the PR region, while 288 were in the RT region and 18 were in the IN region. PS-GRT missed one T69D (frequency level = 31%) and one K70R (frequency level = 26%), which were both NRTI-associated DRMs (Figure 2b). The frequency levels of the detected DRMs are summarized in Table 3.

#### 3.2.4. Accuracy

**Figure 3:**
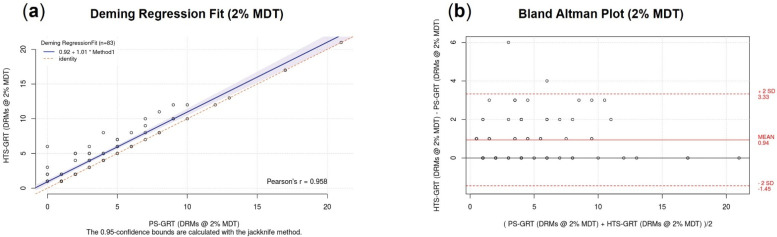

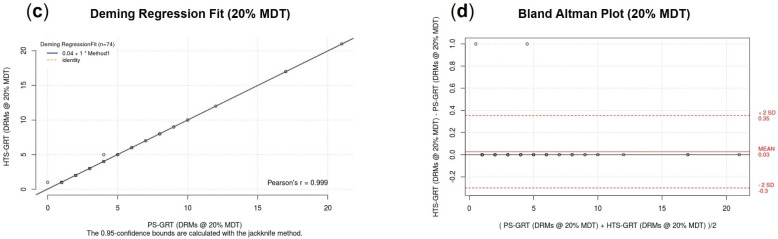
Comparison of PS-GRT and HTS-GRT for drug-resistant mutation detection using Deming regression fits and Bland–Altman plots at the 2% and 20% mutation detection thresholds, respectively: (**a**) linear range of drug-resistant mutation detection at the 2% mutation detection threshold; (**b**) Bland–Altman plot of drug-resistant mutation detection at the 2% mutation detection threshold; (**c**) linear range of drug-resistant mutation detection at the 20% mutation detection threshold; (**d**) Bland–Altman plot of drug-resistant mutation detection at the 20% mutation detection threshold. Each point represents the number of detectable drug resistance mutations using both methods for each sample. The bias is the mean of the difference between the two methods. The 95% limit of agreement accounts for 95% of the possible differences. Note: HTS-GRT, high-throughput-sequencing-based genotypic resistance test; PS-GRT, population-sequencing-based genotypic resistance test; PI, protease inhibitor; NRTI, nucleoside reverse transcriptase inhibitor; NNRTI, non-nucleoside reverse transcriptase inhibitor; IN, integrase; MDT, mutation detection threshold; DRM, drug-resistant mutation.

To demonstrate commutability between the two methods, we compared the numbers of DRMs detected by PS-GRT and HTS-GRT, respectively, for each sample at the 2% and 20% mutation detection thresholds (Figure 3), using Deming regression fits and Bland–Altman plots. At the 2% mutation detection threshold, the numbers of DRMs detected by the two methods for each sample were well correlated (Pearson’s r = 0.958; Figure 3a). In Figure 3b, the Bland–Altman plot shows a mean difference of 0.94. At the 20% mutation detection threshold, a high correlation was observed between the two methods (Pearson’s r = 0.999; Figure 3c). In Figure 3d, Bland–Altman plot shows a mean difference of only 0.03. Generally, the methods were comparable (commutable) in their detection of the DRMs at the 20% mutation detection threshold. When the threshold was lowered to 2%, HTS-GRT displayed higher sensitivity by consistently detecting more DRMs per sample compared to PS-GRT, as evidenced by our findings.

#### 3.2.5. Genotypic Resistance Interpretation

In the present study, 15% (*n* = 15) of the 103 samples showed no resistance to the four antiretroviral drug classes based on PS-GRT. After retesting with HTS-GRT, three showed a change in their genotypic resistance status at the 2% mutation detection threshold. The first sample had an A62V NRTI-associated DRM that was detected at a frequency level of 2%. The second sample had a F77L NRTI-associated DRM and V108I NNRTI-associated DRM that were both detected at a frequency level of 2%. The third sample had a T97A INSTI-associated DRM that was detected at a frequency level of 3%.

PS-GRT identified 88 samples that were resistant to at least one or more antiretroviral drug class(es). Subsequent retesting with HTS-GRT again revealed two samples which harbored resistance to additional drug classes at the 2% mutation detection threshold. One sample was previously found to show resistance to NRTIs and NNRTIs by PS-GRT and was subsequently found to have an additional M46L PI-associated DRM (frequency level = 2%) by HTS-GRT. Another sample was previously found to show resistance to NNRTIs and INSTIs by PS-GRT and was subsequently found to have an additional K219E NRTI-associated DRM (frequency level = 3%) by HTS-GRT.

**Figure 4:**
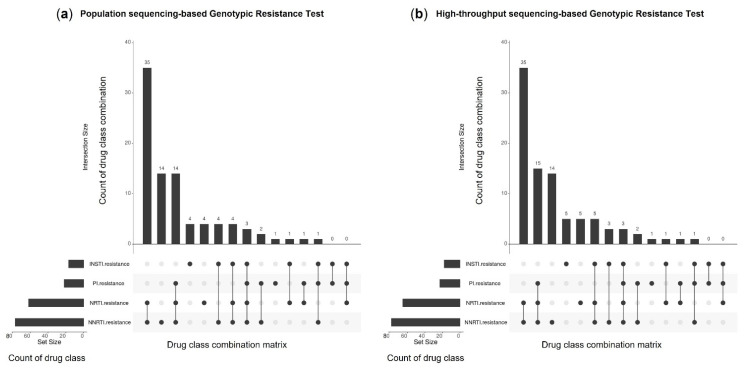
Distribution of the reported genotypic resistance interpretations among the PI, NRTI, NNRTI, and INSTI antiretroviral drug classes detected by: (**a**) population-sequencing-based genotypic resistance test; (**b**) high-throughput-sequencing-based genotypic resistance test. Note: PI, protease inhibitor; NRTI, nucleoside reverse transcriptase inhibitor; NNRTI, non-nucleoside reverse transcriptase inhibitor; IN, integrase.

Figure 4 provides an overview of the reported genotypic resistance interpretations by PS-GRT (Figure 4a) and HTS-GRT (Figure 4b), respectively, across the four antiretroviral drug classes. NNRTI-associated resistance was observed to be the most prevalent, followed by NRTI, PI and INSTI. Using PS-GRT, NRTI/NNRTI two-class resistance accounted for 40% (*n* = 35) of the samples with DRMs, while PI/NRTI/NNRTI three-class resistance and NNRTI one-class resistance accounted for another 32% (*n* = 28). Using HTS-GRT at the 2% mutation detection threshold, NRTI/NNRTI two-class resistance accounted for 38% (*n* = 35) of the samples with DRMs, while PI/NRTI/NNRTI three-class resistance and NNRTI one-class resistance accounted for another 32% (*n* = 29).

## 4. Discussion

In this study, we evaluated the diagnostic performance of a laboratory-developed HTS-GRT using 103 archived clinical samples that were previously tested for HIV drug resistance using population sequencing. For HTS-GRT, we used a 2% mutation detection threshold as a cut-off to identify variants during the manual review of the sequence read alignments in the BAM files. Stoler and Nekrutenko [40] previously reported that the median error rate of the MiSeq system is 0.473%, with a standard deviation of 0.938%. Therefore, the proposed 2% mutation detection threshold for the manual review process seems to be an appropriate cut-off for the purpose of this study.

Overall, a total of 1152 variants were jointly detected by both methods, 339 of which were DRMs. HTS-GRT identified 196 additional variants, 78 of which were clinically actionable DRMs. Using PS-GRT as the reference, HTS-GRT achieved an overall concordance rate of 100% in the DRM detection. On the other hand, using HTS-GRT as the reference at the 2% mutation detection threshold, PS-GRT managed to achieve a good overall concordance rate of 81% (339/417), albeit that it missed a considerable proportion of the DRMs. However, the difference in the number of detected DRMs by the two methods was statistically significant based on McNemar’s test (McNemar’s χ^2^ = 69.014, df = 1, *p*-value < 0.05).

Interestingly, PS-GRT detected 21 variants with frequency levels below 20% (median: 15%; IQR 14–16%), 13 of which were DRMs (median: 15%; IQR 14–16%). The PS-GRT also missed five variants with frequency levels above 20% (median: 26%; IQR 23–31%), two of which were DRMs (median: 28.50%; IQR 27.25–29.75%). To explore this further, we performed a Probit regression analysis using our variant frequency data to estimate the 95% LoD of the PS-GRT. The Probit analysis showed that the 95% LoD ranged from 23 to 28%, which was consistent with the abovementioned findings; i.e., the detection of variants below PS-GRT’s LoD was based on chance due to stochastic effects.

Notwithstanding the multitude of studies which have reported the associations between minority DRMs and treatment failures [25,26,27,28,29,30], the clinical significance of minority DRMs remains debatable. Additional studies are still required in order to establish a clinically actionable mutation detection threshold so as to distinguish minority DRMs that lead to poor virologic suppression. In the present study, a total of 196 variants were missed by PS-GRT, 78 of which were clinically actionable DRMs based on the Stanford HIV Drug Resistance Database. Significantly, K103N [12,41,42,43,44], Y181C [41,44,45], and M184V [41] are the three most frequently reported minority DRMs associated with early treatment failures, highlighting the need for a sensitive method that can be used during baseline drug resistance testing. As evidenced through this validation exercise, using 103 clinical samples, the HTS-GRT identified four K103N, four Y181C, and six M184V which, otherwise, would have been overlooked by the less sensitive PS-GRT, notwithstanding the possibility of the false detection of DRMs by HTS-GRT, a limitation that cannot be ignored. In an attempt to resolve this, we performed a manual review of the BAM files, using IGV to verify that the reads were correctly mapped based on five mapping quality metrics, thereby minimizing the chance of false detection. It is also noteworthy that all the variants detected by HTS-GRT had a median read depth of 7097 (IQR 4301–10,537). The high sequencing depth adds further confidence to these results. We also performed a separate manual review of the Sanger electropherograms using RECall to ascertain whether or not the variants and DRMs were missed by PS-GRT.

We acknowledge that our results should be interpreted considering the following limitations. Firstly, over half of the samples had no viral load information. The testing performance of each sequencing method is deeply affected by the viral load of the sample; thus, the results obtained could be biased. Another limitation of this study is the small number of non-CRF01_AE and non-B subtypes included in the evaluation. Further studies targeting the non-CRF01_AE and non-B subtypes would provide additional insights on how HTS-GRT performs with respect to these uncommon subtypes.

In conclusion, our study complements previous studies [23,24,33,46,47,48,49] that support the notion that the population sequencing and high-throughput sequencing are well correlated. Moreover, using HTS-GRT, we can detect minority DRMs that have previously been reported to be associated with poor virologic suppression. Therefore, the high-throughput sequencing method appears to be an excellent alternative for the clinical management of HIV/AIDS patients in a clinical setting.

## Figures and Tables

**Table 1:** Primers used in the study.

Method	Target Region	Nucleotide Positions ^φ^	Sequence (5′ to 3′)	Orientation	Expected Size
PS-GRT	PR	2071 to 2091	GAR AGA CAG GCT AAT TTT TTA	Forward	1722
	RT	3774 to 3792	CCC ANT CAG GAA TCC AGG T	Reverse	
	IN	4176 to 4204	GGA GGA AAT GAA MAA RTA GAT AAA TTA GT	Forward	885
	IN	5040 to 5060	ACC TGC CAT CTG TTT TCC ATA	Reverse	
HTS-GRT	PR	2071 to 2091	GAR AGA CAG GCT AAT TTT TTA	Forward	2990
	IN	5040 to 5060	ACC TGC CAT CTG TTT TCC ATA	Reverse	

**^φ^** The nucleotide positions were numbered according to the Los Alamos HIV database guidelines, using the GenBank accession number K03455 as the reference. Note: HTS-GRT, high-throughput-sequencing-based genotypic resistance test; PS-GRT, population-sequencing-based genotypic resistance test; PR, protease; RT, reverse transcriptase; IN, integrase.

**Table 2:** Characteristics of the 103 clinical samples.

Characteristics	Count	Median (IQR)
**HIV-1 Viral Load**		
With	44 (43%)	4.80 log 10 copies/mL (4.35–5.20)
Without	59 (57%)	
**HIV-1 Subtype**		
B	20 (19%)	
C	3 (3%)	
G	2 (2%)	
F2	1 (1%)	
CRF01_AE	70 (68%)	
CRF33_01B	2 (2%)	
CRF07_BC	1 (1%)	
CRF48_01B	1 (1%)	
CRF51_01B	1 (1%)	
CRF52_01B	1 (1%)	
CRF54_01B	1 (1%)	

Note: IQR, interquartile range.

**Table 3:** Frequency levels of the detected variants.

Characteristics	Count	Unique Codon Positions	Frequency Level (Median and IQR)	McNemar’s χ^2^ (*p*-Value)
**2% Mutation Detection** **Threshold:**				
***gag*** **-*pol* region**				
HTS-GRT (Detected)	1348	86	100% (80–100%)	
PS-GRT (Detected)	1152	72	100% (99–100%)	**194.01 (<0.05)**
PS-GRT (Missed)	196	68	4% (2–9%)	
**PR**				
HTS-GRT (Detected)	651	29	100% (99–100%)	
PS-GRT (Detected)	604	26	100% (99–100%)	**45.02 (<0.05)**
PS-GRT (Missed)	47	20	3% (2–5%)	
**RT**				
HTS-GRT (Detected)	592	37	99% (27–100%)	
PS-GRT (Detected)	468	36	100% (90–100%)	**122.01 (<0.05)**
PS-GRT (Missed)	124	35	4% (3–10%)	
**IN**				
HTS-GRT (Detected)	105	20	99% (16–100%)	
PS-GRT (Detected)	80	17	99.50% (96.25–100%)	**23.04 (<0.05)**
PS-GRT (Missed)	25	13	5% (2–10%)	
**20% Mutation Detection** **Threshold:**				
***gag*** **-*pol* region**				
HTS-GRT (Detected)	1136	80	100% (99–100%)	
PS-GRT (Detected)	1131	80	100% (99–100%)	3.20 (0.07)
PS-GRT (Missed)	5	3	26% (23–31%)	
**PR**				
HTS-GRT (Detected)	600	27	100% (99–100%)	
PS-GRT (Detected)	600	27	100% (99–100%)	-
PS-GRT (Missed)	0	-	-	
**RT**				
HTS-GRT (Detected)	459	36	100% (92–100%)	
PS-GRT (Detected)	454	36	100% (100%)	3.20 (0.07)
PS-GRT (Missed)	5	3	26% (23–31%)	
**IN**				
HTS-GRT (Detected)	77	17	100% (98–100%)	
PS-GRT (Detected)	77	17	100% (98–100%)	-
PS-GRT (Missed)	0	-	-	

Note: HTS-GRT, high-throughput-sequencing-based genotypic resistance test; PS-GRT, population-sequencing-based genotypic resistance test; PR protease; RT, reverse transcriptase; IN, integrase; IQR, interquartile range.

**Table 4:** Frequency levels of the detected drug resistance mutations.

Characteristics	Count	Unique Codon Positions	Frequency Level (Median and IQR)	McNemar’s χ^2^ (*p*-Value)
**2% Mutation Detection** **Threshold:**				
***gag*** **-*pol* region**				
HTS-GRT (Detected)	417	57	100% (80–100%)	
PS-GRT (Detected)	339	50	100% (92–100%)	**76.01 (<0.05)**
PS-GRT (Missed)	78	30	5% (3–11%)	
**PR**				
HTS-GRT (Detected)	24	13	100% (99–100%)	
PS-GRT (Detected)	21	11	100% (99–100%)	1.33 (0.25)
PS-GRT (Missed)	3	3	3% (2–3.50%)	
**RT**				
HTS-GRT (Detected)	371	33	99% (29–100%)	
PS-GRT (Detected)	300	30	100% (91–100%)	**69.01 (<0.05)**
PS-GRT (Missed)	71	24	5% (3–11%)	
**IN**				
HTS-GRT (Detected)	22	11	99% (45.75–100%)	
PS-GRT (Detected)	18	11	100% (98.25–100%)	2.25 (0.13)
PS-GRT (Missed)	4	3	10.50% (8.25–11%)	
**20% Mutation Detection** **Threshold:**				
***gag*** **-*pol* region**				
HTS-GRT (Detected)	328	53	100% (95.75–100%)	
PS-GRT (Detected)	326	52	100% (96–100%)	0.50 (0.48)
PS-GRT (Missed)	2	2	28.50% (27.25–29.75%)	
**PR**				
HTS-GRT (Detected)	20	11	100% (99–100%)	
PS-GRT (Detected)	20	11	100% (99–100%)	-
PS-GRT (Missed)	0	-	-	
**RT**				
HTS-GRT (Detected)	290	31	100% (94–100%)	
PS-GRT (Detected)	288	30	100% (94.75–100%)	0.50 (0.48)
PS-GRT (Missed)	2	2	28.50% (27.25–29.75%)	
**IN**				
HTS-GRT (Detected)	18	11	100% (98.25–100%)	
PS-GRT (Detected)	18	11	100% (98.25–100%)	-
PS-GRT (Missed)	0	-	-	

Note: HTS-GRT, high-throughput-sequencing-based genotypic resistance test; PS-GRT, population-sequencing-based genotypic resistance test; PR, protease; RT, reverse transcriptase; IN, integrase; IQR, interquartile range.

## Data Availability

The data presented in this study are available on request from the corresponding author. The data are not publicly available due to privacy and ethical concerns.
